# Nivolumab for Previously Treated Patients with Non-Small-Cell Lung Cancer—Daily Practice versus Clinical Trials

**DOI:** 10.3390/jcm9072273

**Published:** 2020-07-17

**Authors:** Magdalena Knetki-Wróblewska, Dariusz M. Kowalski, Maciej Krzakowski

**Affiliations:** Department of Lung Cancer and Chest Tumors, Maria Sklodowska-Curie National Research Institute of Oncology, 02-781 Warsaw, Poland; Dariusz.Kowalski@pib-nio.pl (D.M.K.); Maciej.Krzakowski@pib-nio.pl (M.K.)

**Keywords:** non-small-cell lung cancer, nivolumab, Expanded Access Program, real-world data, daily practice, prognostic factors

## Abstract

Based on the results of the CheckMate 017 and CheckMate 057 studies, nivolumab therapy has become a new standard treatment for both squamous and non-squamous non-small-cell lung cancer (NSCLC). However, due to the specific inclusion criteria of these clinical trials, the efficacy and safety of nivolumab in real-world practice were not certain. In general, the real-world results of nivolumab treatment have been consistent with those obtained in clinical trials. Additional analyses of the real-world data have made the identification of prognostic factors possible. Good performance status is the most significant predictor of clinical benefit. Brain metastases, liver metastases, *EGFR* mutation, malignant pleural effusion, and a high number of metastatic sites were identified as negative prognostic factors. By contrast, a longer time to disease progression (>6 months) from the beginning of prior chemotherapy and an objective response to chemotherapy seem to have positive prognostic value in the case of nivolumab treatment. In terms of patient age, the data are inconclusive. Some blood biomarkers can also be considered significant prognostic factors.

## 1. Introduction

Immune checkpoint inhibitors targeting programmed death 1 (PD-1) and its ligand (PD-L1) have significantly changed the management of advanced non-small-cell lung cancer (NSCLC) in recent years [1]. Nivolumab, a fully human antibody directed against PD-1, has been approved for previously treated advanced NSCLC. Nivolumab was associated with significantly longer overall survival (OS) than docetaxel and had a good safety profile in squamous and non-squamous NSCLC in two pivotal phase III clinical trials (CheckMate 017 and CheckMate 057) [2,3]. Pooled analysis of long-term outcomes confirmed the significant clinical efficacy of nivolumab compared with that of docetaxel [4,5]. The value of nivolumab was assessed in prospective clinical trials, the results of which were required for drug registration. However, further real-world NSCLC population studies and evaluation of the value of nivolumab in clinical practice are necessary to select a subgroup of patients in whom clinical benefits are most likely. The patient population is much more diverse in clinical practice than in clinical trials. Negative prognostic factors are frequent issues in many cases, with poor performance status, brain or liver metastases, and elderly age being the most common. Real-world data can also be used to identify additional prognostic factors that may be helpful in treatment decision-making. Some real-world data concerning nivolumab have recently been published, including those derived from the Expanded Access Program (EAP) and post-registration studies (data for nivolumab are more frequently published than data for atezolizumab and pembrolizumab). This paper aims to provide an overview of the selected real-world studies on nivolumab and to describe predictive factors of value in clinical practice.

## 2. Nivolumab in Clinical Trials

The CheckMate 057 trial was designed for patients with non-squamous NSCLC. Eligible patients had primary CS IIIB/IV NSCLC or recurrent NSCLC after radiation therapy or surgical resection and documented disease progression during or after one platinum-based doublet chemotherapy regimen [3]. Patients with an acceptable general condition and adequate organ function without major comorbidities were included [3]. In all, 582 patients were randomized: 292 were assigned to receive nivolumab at a dose of 3 mg/kg every 2 weeks, and 290 to receive docetaxel at a dose of 75 mg/m^2^ every 3 weeks. OS was the primary endpoint. The key secondary endpoints were investigator-assessed confirmed objective response rate and progression-free survival (PFS). Tumor response was assessed with the use of the Response Evaluation Criteria in Solid Tumors, version 1.1 (RECIST 1.1), at week 9 and then every 6 weeks until disease progression. Safety was assessed with the Cancer Institute Common Terminology Criteria for Adverse Events (CTCAE), version 4.0.

The median OS was longer in the nivolumab group than in the docetaxel group (12.2 months, 95% confidence interval (CI) 9.7–15.0, versus 9.4 months, 95% CI 8.1–10.7; hazard ratio (HR) 0.73, 95% CI 0.59–0.89; *p* = 0.002). The objective response rate was 19% with nivolumab versus 12% with docetaxel (*p* = 0.02). Treatment-related adverse events (AEs) of any grade were reported in 69% of patients in the nivolumab group and in 88% in the docetaxel group, while grade 3–4 AEs occurred in 10% of the nivolumab group and in 54% of the docetaxel group [3]. The most important data from that study are presented in Table 1.

The CheckMate 017 trial was designed for patients with squamous NSCLC. Eligible patients had CS IIIB/IV NSCLC and documented disease progression after one platinum-based doublet chemotherapy [4]. The main inclusion or exclusion criteria and treatment outline were similar to those for the CheckMate 057 trial. In all, 272 patients underwent randomization: 135 patients were assigned to receive nivolumab and 137 to receive docetaxel [4].

The median OS was longer with nivolumab than with docetaxel (9.2 months, 95% CI 7.3–13.3, versus 6.0 months, 95% CI 5.1–7.3; HR 0.59, 95% CI 0.44–0.79; *p* < 0.001). The response rate was 20% with nivolumab versus 9% with docetaxel (*p* = 0.008). Treatment-related AEs of any grade occurred in 58% of patients in the nivolumab group and in 86% in the docetaxel group. Grade 3–4 AEs occurred in 7% of the nivolumab group and in 55% of the docetaxel group. The most important data from that study are presented in Table 1.

A pooled analysis of long-term outcomes confirmed the efficacy of nivolumab [4,5]. The 4-year OS rate was 14% in patients treated with nivolumab, compared with 5% in patients treated with docetaxel (14.9% for patients with non-squamous NSCLC and 9.4% for patients with squamous NSCLC in the nivolumab population); the 5-year OS rate was 13.4% with nivolumab versus 2.6% with docetaxel (HR 0.68, 95% CI 0.59–0.78) [4,5]. Patients in the nivolumab group who achieved an objective response had the best long-term results. Median OS in patients with an objective response was not reached in the nivolumab group (95% CI 25.6–not reached) versus 17.1 months (95% CI 11.1–28.7) in the docetaxel group. The 4-year OS rate in patients with an objective response was 58% with nivolumab and 12% with docetaxel [4].

## 3. Nivolumab in Daily Practice

Patients likely to participate in clinical trials have to meet strictly defined, challenging criteria. Inclusion criteria usually only allow for the treatment of patients with Eastern Cooperative Oncology Group (ECOG) 0–1, without significant comorbidities, and with normal laboratory results. However, in clinical practice, the population of pretreated patients with NSCLC is very diverse. Poor performance status (ECOG 2–3), brain metastases, liver metastases, and elderly age, as well as rapid disease progression after chemotherapy are the most common problems. Real-world data can be used to assess the efficacy of nivolumab in clinical practice. Some real-world data have recently been published, including those derived from the EAP, which made it possible for many patients to be treated before the medicine was reimbursed in their countries. In total, data regarding 4800 patients have been published.

### 3.1. Efficacy

The Italian cohort is the largest reported group from the nivolumab EAP [6,7]. Inclusion criteria included CS IIIB/IV NSCLC, ECOG 0–2, adequate organ function, life expectancy of at least 6 weeks, and progression after at least one line of systemic treatment for advanced or metastatic disease. Patients with progression within 6 months after radical treatment for locally advanced disease were also eligible. Unstable brain metastases and active known or suspected autoimmune disease (with some exceptions) were contraindications for nivolumab treatment. For 1588 patients with non-squamous NSCLC, the median OS was 11.3 months, the 1-year OS rate was 48%, and the median PFS was 3.0 months, with a 1-year PFS rate of 22% [6]. The median OS was 7.9 months and the 1-year OS rate was 39% in the 371 patients with squamous NSCLC [7]. The overall response rate (ORR) was 18% for both the squamous and non-squamous NSCLC patient groups. Similar efficacy data from other countries are also available. In a group of 901 Japanese patients treated in an observational post-registration study, the median OS was 14.6 months for the entire patient population and the 1-year OS rate was 54.3% [8]. The median OS was 15.1 months for patients with non-squamous NSCLC and 12.3 months for patients with squamous NSCLC [8]. The median PFS for the entire patient population was 2.1 months and the ORR was 20.5% [8]. Survival and ORR data reported in other publications (patients in the EAP and in routine clinical practice) are summarized in Table 2.

### 3.2. Prognostic Factors in Real-World Practice

The clinical benefit of nivolumab, as shown in registration trials, applies to the entire patient population, but further analysis of the data suggests that some patients may benefit more than others. However, unfavorable responses to nivolumab treatment can be also observed. Therefore, it is important to identify additional prognostic factors that can be used in treatment decision-making. The real-world data are helpful in this regard.

#### 3.2.1. Performance Status

Performance status is a crucial factor in treatment decision-making for patients with NSCLC. ECOG 0–1 is required in clinical trials, but the patient population is much more diverse in clinical practice. According to the real-world data, the prevalence of patients with ECOG ≥ 2 who are treated with nivolumab ranges from 3% to 46% [6,7,8,9,10,11,12,13,14,15]. The prognostic value of performance status has been well documented. 

Multivariate survival analysis in the Italian EAP cohort of patients with non-squamous NSCLC showed that ECOG 2 performance status is an independent prognostic factor for early death (*p* < 0.0001) [6]. Poor performance status (ECOG 2), compared with ECOG 0, was also identified as an independent prognostic factor for death in the Italian EAP squamous NSCLC cohort (HR 2.76, 95% CI 1.65–4.62; *p* < 0.0001) [7]. In this cohort, the risk of death was also higher in patients with ECOG 1 than in patients with ECOG 0 (HR 1.57, 95% CI 1.17–2.11; *p* = 0.003) [7]. Similar results were obtained in the analysis of a group of 901 Japanese patients, in which 17.4% of the patients had an ECOG score of 2, 3, or 4 (HR 0.39, 95% CI 0.51–0.8; *p* < 0.0001) [8]. Poor performance status was also a risk factor for short PFS (HR 0.64, 95% CI 0.51–0.8; *p* < 0.0001) [8]. Multivariate analysis of the Portuguese EAP data identified performance status as the only independent prognostic factor (*p* < 0.0006) [13]. In a univariate analysis, the risk of death was much lower in patients with ECOG 0–1 than in patients with ECOG 2 (HR 3.8, 95% CI 2.3–6.07; *p* < 0.0001) [13]. Another univariate analysis reported an OS of 3.4 months (95% CI 2.3–4.4) in patients with ECOG 2 versus 11.79 months (95% CI 8.5–15.07) in patients with ECOG 1. The median OS for patients with ECOG 0 was not reached [14]. Some data related to the prognostic value of performance status are summarized in Table 3.

#### 3.2.2. Liver Metastases

A pooled analysis of the CheckMate 017 and CheckMate 057 trials with updated results from more than 3 years of follow-up included a subgroup analysis of patients with liver metastases [16]. Liver metastases were found in 23% of 854 patients at baseline. Although nivolumab had a confirmed OS benefit in patients with liver metastases (HR 0.68, 95% CI 0.50–0.91), the median OS for patients with liver metastases was 6.8 months in the nivolumab group and 5.9 months in the docetaxel group (HR 0.68, 95% CI 0.50–0.91), while for the entire patient population the median OS was 11.1 months for the nivolumab group and 8.1 months for the docetaxel group (HR 0.70, 95% CI 0.61–0.81) [16]. Patients with and without liver metastases were not directly compared.

Liver metastases were determined to be an independent negative prognostic factor for OS in multivariate analyses of some real-world data [6,7,8]. For Italian patients with squamous NSCLC, the HR was 1.44 (95% CI 1.04–1.98; *p* = 0.03) [7], and for non-squamous NSCLC the odds ratio (OR) for early death was 0.47 (95% CI 0.35–0.61; *p* < 0.0001) [6]. However, in another publication, the negative prognostic value of liver metastases was not confirmed [9]. In a retrospective analysis of 215 patients with NSCLC who received nivolumab, atezolizumab, or pembrolizumab (19.1% of patients had liver metastases), there was a higher risk of death in patients with liver metastasis than in those without (HR 2.04, 95% CI 1.33–3.13) [17]. Additional negative prognostic factors for patients with NSCLC and liver metastases were low albumin level, poor performance status, driver mutation, and having five or more liver metastases. 

#### 3.2.3. Brain Metastases

About 10% of non-oncogene addicted patients with NSCLC have brain metastases at diagnosis and 25–40% develop brain metastasis during the course of the disease. A pooled analysis of the CheckMate 017 and CheckMate 057 trials showed that 11% of the included patients had brain metastasis at baseline, but no detailed information about the intracranial efficacy of nivolumab were provided in the primary publications [4]. However, nivolumab was more effective than docetaxel in terms of OS in the entire analyzed patient population [4]. CheckMate 012 (NCT01454102) was a phase I, multicohort study evaluating nivolumab alone or in combination with other therapies for the treatment of patients with advanced NSCLC and untreated brain metastases (12 patients, arm M) [18]. Intracranial response was evaluated with magnetic resonance imaging [18]. The ORR was 16.7% (two patients) in that small study group; however, progressive disease was observed in the majority of patients [18].

Nivolumab therapy is routinely used in patients who have undergone primary resection or irradiation for brain metastases and whose clinical condition improved after receiving local treatment. Retrospective analyses of real-world data showed that nivolumab has intracranial activity [19,20,21]. Twenty-six percent of patients with non-squamous NSCLC in the Italian EAP had asymptomatic or controlled brain metastases [19]. The disease control rate was 40% and the ORR was 17%. The median OS in patients with asymptomatic or controlled brain metastases was 8.6 months (95% CI 6.4–10.8) compared with 11.3 months (95% CI 10.2–12.4) for the entire cohort [20]. In the cohort with squamous NSCLC, 10% of 372 patients had asymptomatic brain metastases. The median OS was 5.8 months (95% CI 1.9–9.8) [21]. A direct comparison of the efficacy of nivolumab in patients with and without brain metastases showed significant differences in OS [14]. In a group of 188 patients, 22% had brain metastases. The median OS was 5.09 months (95% CI 0.3–9.8) in the patients with brain metastases versus 14.8 months (95% CI 11.5–17.3) in patients without brain metastases [14]. In another cohort, in which 14.8% of 472 patients had brain metastases, the median OS reached 9 months (95% CI 5.5–13.3) in patients with brain metastases and 13.1 months (95% CI 11.5–17.1; *p* = 0.007) in patients without brain metastases [12]. Some studies have identified brain metastases as an independent negative prognostic factor [8], but others have not [6,7,9,10].

#### 3.2.4. Elderly Patients

More than 40% of patients in the CheckMate 017 and CheckMate 057 populations were over 65 years of age, including about 7% of patients who were over 75 years of age [2,3]. Nivolumab was effective in the whole group, although for patients over 75 years of age the clinical benefit was uncertain (HR 0.9; 95% CI 0.43–1.87) [3]. The findings of the phase II CheckMate 171 trial have been published recently [22]. Overall, 811 patients with previously treated advanced squamous NSCLC were included, of whom 278 were aged over 70 years and 125 were aged over 75 years [22]. The median OS was similar in all age groups: 10.0 months (95% CI 9.2–11.2) in all patients, 10.0 months (95% CI 8.3–11.4) in those aged over 70 years, and 11.2 months (95% CI 7.9–14.2) in those aged over 75 years. The safety profile was similar across age-determined populations; however, low-grade diarrhea was more common in patients over 70 years of age than in those aged 70 or younger [22]. AEs were reported in 13.9% of all patients, in 15.8% of those aged over 70 years, and in 18.4% of those aged over 75 years [22]. In an Italian population of 371 patients with squamous NSCLC, OS was reduced in patients aged 75 years or older (5.8 months, 95% CI 3.5–8.1) versus patients aged under 65 years (8.6 months, 95% CI 5.2–11.9), patients aged 65 to less than 75 years (8.0 months, 95% CI 5.6–10.4), and the overall population (7.9 months, 95% CI 6.2–9.6) [23]. Discontinuation rates due to treatment-related AEs were low irrespective of age (4–5%) [24]. However, a retrospective analysis of 324 Belgian patients with NSCLC showed no significant difference between older (≥70) and younger (<70 years) patients in terms of PFS (4 months versus 3.7 months, *p* = 0.483) and OS (9.3 months versus 8.4 months, *p* = 0.638) [25]. The incidence of AEs of all grades and of grade 3–4 AEs was also similar between age groups [25]. Similarly, in a group of Italian patients with non-squamous NSCLC, 522 of 1588 patients were over 70 years of age; these patients reached a median OS of 11.5 months (95% CI 10.0–13.0), while for the 232 patients aged over 75 years OS was 12.0 months (95% CI 9.2–14.8) [6]. There were no significant differences in the incidence of treatment-related AEs in the subgroups defined by age (6–7% of AEs were grade 3–4) [6]. Some studies have confirmed that treatment outcomes in clinical practice are not affected by age [11,12,26,27], whereas others have reported nivolumab treatment to have less favorable results in patients aged over 75 years [9].

#### 3.2.5. *EGFR* Status

Of the patients in the CheckMate 057 trial, 15% had an *EGFR* mutation. Nivolumab was not better than docetaxel in that subset of patients (HR 1.18, 95% CI 0.69–2.0) [3].

In an Italian cohort of patients with non-squamous NSCLC, 102 patients (6.4%) had an *EGFR* mutation [28]. No statistically significant difference in OS was observed in patients with an *EGFR* mutation versus that in those without. OS reached 11 months in patients with *EGFR* wild-type tumors versus 8.3 months in patients with *EGFR*-mutant tumors (HR 1.11, 95% CI 0.84–1.47; *p* = 0.4) [28]. A study by the Galician Lung Cancer Group showed that OS was higher in patients without an *EGFR* mutation than in patients with *EGFR*-mutated NSCLC (12.8 versus 4.8 months, *p* = 0.12) [14]. Although univariate (HR 1.11, 95% CI 0.84–1.45; *p* = 0.46) and multivariate (HR 1.13, 95% CI 0.82–1.56; *p* = 0.45) analysis of 901 Japanese patients (12.9% with *EGFR* mutation) failed to determine the prognostic value of *EGFR* mutation [8], most reports are in line with the CheckMate 057 results and confirmed the negative prognostic value of *EGFR* mutation in patients treated with nivolumab. OS in 25 Canadian patients with *EGFR* mutation was 3.38 months, while in 229 patients with wild-type *EGFR* it was 13.37 months (HR 2.32, 95% CI 1.37–3.93; *p* = 0.002) [12]. A multivariate analysis of 613 patients (15% of whom had an *EGFR* mutation) showed *EGFR* mutation or *ALK* translocation to have negative prognostic value in terms of PFS (HR 1.45, 95% CI 1.12–1.86) [26].

#### 3.2.6. Sensitivity to Previous Chemotherapy

A post hoc exploratory multivariate analysis of the CheckMate 057 population suggested that some patients might be at a higher risk of death within the first 3 months of treatment. The following known negative prognostic factors were considered: less than 3 months since last treatment, progressive disease as best response to prior treatment, and an ECOG score of 1 [29].

Real-life experience with nivolumab has shown that sensitivity to previous chemotherapy could have prognostic value. The Netherlands Cancer Institute published the results of 248 patients treated with nivolumab [10]. Of the 189 patients who had a documented response to prior platinum-based doublet therapy, 38.6% had progressive disease as the best response. OS was 13.1 months in patients who had been sensitive to the chemotherapy and only 5.0 months in chemotherapy-refractory patients (HR 1.7, 95% CI 1.108–2.642; *p* = 0.015). An analysis of 221 patients showed that time to progression could also have prognostic value [11]. Patients who had disease progression within 6 months of platinum therapy did worse than those who had a longer PFS than 6 months on platinum therapy (3.7 months versus 11.8 months; HR 0.39, 95% CI 0.26–0.6; *p* < 0.0001) [11]. The positive prognostic value of both ORR and PFS of more than 6 months since the beginning of prior chemotherapy was presented in another publication [30].

### 3.3. Safety

In the CheckMate 057 study, the frequency of any-grade AEs related to nivolumab treatment was 69%, while the frequency of grade 3–4 AEs was 10% [3]. The most common any-grade AEs were fatigue (16%), nausea (12%), decreased appetite (10%), and asthenia (10%). The rate of discontinuation due to nivolumab-related AEs was 5% [3]. In the CheckMate 017 study, any-grade AEs were reported in 58% of patients, while grade 3–4 AEs were reported in 7% of patients, and treatment was discontinued due to nivolumab-related AEs in 3% of patients [2]. The safety profile established in clinical practice seems to be consistent with that determined in clinical trials. The relevant data are summarized in Table 4. The differences in the frequency of any-grade AEs between some publications could be associated with less precise reporting of AEs outside clinical trials.

## 4. Summary

Based on the results of the CheckMate 017 and CheckMate 057 studies, nivolumab therapy has become a new standard of care for both squamous and non-squamous NSCLC. Due to the specific inclusion criteria of the clinical trials, the efficacy and safety of nivolumab in real-world practice were not certain. However, some data from the EAP and from post-registration studies have recently been published, which allows for further evaluation.

In general, the real-world results are consistent with those obtained in clinical trials. From a practical point of view, the important question is how to select a subgroup of patients in whom clinical benefits are most likely. Additional analyses of the real-world data made the identification of prognostic factors possible. 

Performance status is the most important prognostic factor. Several multivariate analyses showed ECOG 0–1 to be the most significant predictor of clinical benefit [6,7,13,30,31]. An analysis that focused on negative prognostic factors in response to nivolumab therapy clearly identified the following risk factors of early death: ECOG ≥ 2 (OR 5.66, 95% CI 2.01–15.61; *p* < 0.001), C-reactive protein to albumin ratio >0.3 (OR 10.56, 95% CI 3.61–3086; *p* < 0.001), and poor response to first-line chemotherapy (OR 2.06, 95% CI 1.03–4.14; *p* < 0.001) [31]. Additionally, many authors suggest that liver metastases, brain metastases, and *EGFR* mutation are negative prognostic factors associated with a higher risk of death. Malignant pleural effusion and a high number of metastatic sites were also identified as negative prognostic factors [30,32]. By contrast, a longer PFS on platinum therapy (>6 months) and an objective response to chemotherapy seem to have positive prognostic value in the case of nivolumab treatment. In terms of patient age, the data are inconclusive.

Blood biomarkers can also be considered in treatment decision-making. The use of the lung immune prognostic index (LIPI) based on the baseline derived neutrophil to lymphocyte ratio (dNLR) and lactate dehydrogenase (LDH) was suggested [33,34]. A high LIPI value was indicated as an independent negative prognostic factor (HR 3.67, 95% CI 1.96–6.86; *p* < 0.0001) [33]. Several other inflammatory-related markers, such as the neutrophil to lymphocyte ratio (NLR), dNLR, LDH, interleukin 8, and indoleamine 2,3 dioxygenase activity were also found to be important [35,36].

It is noteworthy that some of these prognostic factors are also relevant to chemotherapy [37,38]. The negative prognostic value of parameters such as poor performance status, liver or brain metastases, the number of metastatic sites, and an elevated leukocyte count was demonstrated [37,38].

To summarize—the efficacy and safety of nivolumab in the second-line setting of advanced NSCLC have been established in clinical trials and confirmed in real-world practice. Long-term clinical benefit can be obtained in some patients. Good performance status (ECOG 0–1) is crucial, but other clinical variables such as site and number of metastatic lesions, time to failure of first-line chemotherapy, chemotherapy response status, and specific laboratory results should also be considered. There is a further need to collect data on the efficacy of immunotherapy in real-world clinical practice.

## Figures and Tables

**Table 1:** Nivolumab for previously treated non-small-cell lung cancer—CheckMate 017/057 [2,3].

	CheckMate 017			CheckMate 057		
	Nivolumab	Docetaxel	HR	Nivolumab	Docetaxel	HR
Number of patients	135	137		292	290	
ORR (%)	20	9	2.6; *p* = 0.008	19	12	*p* = 0.02
PFS (months)	3.5	2.8	0.62; *p* < 0.001	2.3	4.2	0.92; *p* = 0.39
OS (months)	9.2	6.0	0.59; *p* < 0.001	12.2	9.4	0.73; *p* = 0.002
AE (any grade, %)	58	86		69	88	
AE (grade 3–4, %)	7	55		10	54	

HR—hazard ratio, ORR—overall response rate, PFS—progression-free survival, OS—overall survival, AE—adverse event.

**Table 2:** Survival in patients treated with nivolumab in real-world practice.

	Number of Patients	OS all Patients (Months)	OS Non-Squamous (Months)	OS Squamous (Months)	PFS (Months)	ORR (%)
Grossi [6]	1588	11.3	11.3	na	3	18
Crino [7]	371	7.9	na	7.9	nd	18
Morita [8]	901	14.6	15.1	12.3 **	2.1	20.5
Dudnik [9]	260	5.9	Squamous vs. non-squamous HR 1.12; *p* = 0.61		2.8	35 *
Schouten [10]	248	10.0	7.8	NR	2.6	21.8
Almazán [11]	221	9.7	12.8	6.9	5.3	17.6
Juergens [12]	472	12.0	11.8	13.1 **	3.5	nd
Figueiredo [13]	229	13.2	Squamous vs. non-squamous HR 0.72; *p* = 0.14		4.9	22.4
Manrique [14]	188	12.85	11.7	14.8 **	4.83	25.5
Brustugun [15]	58	11.7	nd	nd	4.0	nd

OS—overall survival, PFS—progression-free survival, ORR—overall response rate, NR—not reached, na—not applicable, nd—no data, HR—hazard ratio. * 49/260 patients were evaluated for response, ** Statistically non-significant.

**Table 3:** Prognostic value of performance status in patients treated with nivolumab—real-world data.

	% of Patients ECOG ≥ 2	OS (Months)	
		ECOG 0–1	ECOG ≥ 2
Manrique [14]	10	11.79	3.4
Juergens [12]	8.9	12.91	6.77
Almazán [11]	13.6	12.8	2.9
Crino [7]	6	-	HR 2.76 * (2 vs. 0)
Figueiredo [13]	13.2	-	HR 3.8 * (≥2 vs. 0–1)
Schouten [10]	16.1	12.5	4.5
Dudnik [9]	46	9.5	3.5

OS—overall survival, ECOG—Eastern Cooperative Oncology Group, HR—hazard ratio. * Multivariate analysis.

**Table 4:** Incidence of adverse events (AEs) in patients treated with nivolumab—real-world data.

	All (%)	Grade 3–4 (%)	Discontinuation of Therapy Due to an AE (%)
Grossi [6]	32	6	5
Manrique [14]	78	4.8	4.8
Schouten [10]	18	6	nd
Dudnik [9]	62	7	3.5
Crino [7]	29	6	9
Garassino [28]	33	6	2.6
Kobayashi [27]	45	13.3	nd
Figueiredo [13]	76	nd	16

nd—no data.
